# Establishment in an Introduced Range: Dispersal Capacity and Winter Survival of *Trissolcus japonicus*, an Adventive Egg Parasitoid

**DOI:** 10.3390/insects10120443

**Published:** 2019-12-11

**Authors:** David M. Lowenstein, Heather Andrews, Richard J. Hilton, Clive Kaiser, Nik G. Wiman

**Affiliations:** 1 Macomb County Extension Services, Michigan State University, Clinton Township, MI 48036, USA; 2 North Willamette Research and Extension Center, Oregon State University, Aurora, OR 97002, USA; heather.andrews@oregonstate.edu (H.A.); nik.wiman@oregonstate.edu (N.G.W.); 3 Southern Oregon Research and Extension Center, Oregon State University, Central Point, OR 97502, USA; richard.hilton@oregonstate.edu; 4 Hermiston Agricultural Research and Extension Center, Oregon State University, Hermiston, OR 97838, USA; clive.kaiser@oregonstate.edu

**Keywords:** biological control, invasion, overwinter, stink bug, raspberry, stink bug

## Abstract

The herbivorous brown marmorated stink bug, *Halyomorpha halys*, has spread globally, and one of its key parasitoids, *Trissolcus japonicus*, has recently been detected in the pest’s introduced range. For an exotic natural enemy to impact its targeted host in a novel environment, it must disperse, locate hosts, and potentially be redistributed to susceptible sites. Through intentionally releasing *T. japonicus* across four Oregon eco-regions, we investigated an introduced parasitoid’s dispersal capacity in urban sites and in two perennial crops, hazelnut and raspberry. In a second paired field and laboratory study, we investigated *T. japonicus* survival in different plant materials. Within three days of release, adult *T. japonicus* located host egg masses at 45% of sites and, one year later, were detected at 40% of release sites. Areas where released wasps survived winter were mostly urban or semi-natural. In commercial crop release experiments, we recovered the highest percentage of wasps in raspberry within 5 m of the release site but found no statistical difference in dispersal distance with some wasps dispersing up to 50 m. Adult parasitoids survived up to 16 weeks outdoors in the winter, with greater survival over time in bark compared to leaf litter. Wasp survival remained above 50% over the course of a simulated winter environment without precipitation. Our work affirms the continuation of *H. halys* parasitism by *T. japonicus* in novel environments and provides insight into the high population sizes necessary to survive winter and locate host egg masses the following season.

## 1. Introduction

Invasive species continue to arrive in new habitats worldwide [1,2]. The entry of exotic pest species threatens existing crop production [3], forestry [4], and plant communities in urban areas [5]. Native arthropods and trophic webs are also affected by invasive species [6,7,8] through attempting and failing to reproduce in evolutionary traps [9] or displacement [10]. Costs of invasive species management and damage in the United States top 40 billion dollars [11], yet eradication and control efforts require more than financial resources to mitigate their effects on the environment and native species. The accidental entry and intentional release of non-native natural enemies connected to their invasive hosts [12,13,14,15] may contribute to pest suppression through biological control. When both the pest and natural enemy are non-native, the goal of classical biological control is for this ecological host specificity to continue in an introduced environment.

Natural enemies can provide temporary and immediate management of pests through augmentation control or more permanent management through classical biological control. Upon receiving approval to release an exotic natural enemy, several factors must be considered for successful establishment. Where should the natural enemy be released? What population size of the natural enemy should be released to reduce the pest population? How far will the natural enemy disperse in search of hosts? The latter two questions are particularly relevant for efforts that involve minute parasitoid wasps. Careful research into the above factors has led to the commercial availability of at least 100 species of natural enemies for biological control [16]. Genera of Hymenoptera most suitable for redistribution in large population sizes include *Trichogramma* or *Copidosoma*, which exhibit polyembrony [17,18]. While this evolutionary adaptation enables quick regeneration in a laboratory or field environment, the life cycles of wasps who lay a single egg per host are dependent on availability of sufficient host population sizes. The necessary resources for rearing both parasitoid and pest can restrict population sizes available for releases that immediately lower a pest population [16]. However, classical biological control can reduce arthropod pest populations when insecticide control is not practical due to public health concerns or high costs. When classical biological control is effective, adult wasps survive a release from rearing containers or substrate, disperse from the release site, locate host eggs, and establish populations for multiple years [19].

The brown marmorated stink bug, *Halyomorpha halys* (Hemiptera: Pentatomidae) (Stål), is a global polyphagous pest [20] with ongoing biological control programs using the egg parasitoid *Trissolcus japonicus* (Hymenoptera: Platygastridae) (Ashmead) in North America and Europe. Despite being reared in secured quarantine facilities, *T. japonicus* arrived independently to North America multiple times [13,21] and is now present in 12 U.S. States [22] and British Columbia, Canada [23]. Each geographical region is in the early stage of planning redistribution efforts to assist the spread of *T. japonicus*. Laboratory tests of adventive wasps [24,25] indicate comparable *H. halys* parasitism rates to the 70% achieved in its native range [26]. At present, there is minimal guidance for release practices that improve *T. japonicus* establishment and the discovery of host eggs. Platygastridae, which include *Trissolcus* species, can effectively parasitize their intended host insects when released in large populations through inoculative or augmentative biocontrol programs [27]. For example, *Trissolcus basalis* (Wollaston) were released at rates of 12,500–15,000 adults per hectare in order to manage *Nezara viridula* (L.) in tree fruit and soybeans [28,29]. The release of a half million *Trissolcus semistratus* Nees, as parasitized eggs in Turkish wheat fields improved the management of *Eurygaster integriceps* Puton, 1881 (Hemiptera: Scutelleridae) from 25% to 47% in four years [30]. Details on release size, dispersal, and host-recovery in the short and long-term are necessary to evaluate the effect of *T. japonicus* on its intended host as well as on the native Platygastridae community.

### 1.1. Parasitoid Release and Dispersal Assessment

Non-crop vegetation provides refuge from insecticides and farm management practices that disturb habitat. Refugia with wildflowers adjacent to fruit crops may reduce pest damage and benefit parasitoid diversity [31,32]. Minute parasitoids often disperse small distances unless carried by prevailing winds, which makes proximity of floral resources critical for wasps whose activity coincides with host life stages in crops. Parasitoid dispersal can be measured directly through mark-recapture efforts that involve fluorescent dyes [33], albumin-based compounds that are evaluated, with ELISA protein markers [34], or release and recovery through sentinel eggs or sticky cards [29,35] or on the natural host [36]. Low recovery rates are a detriment of mark-recapture studies. Nonetheless, recovered wasps offer valuable insight into movement patterns such as Mymaridae dispersal of 47 m per day [37] and increased *N. viridula* parasitism rates at the closest locations to *Ooencyrtus submetallicus* (Howard, 1897) release [38]. Mark-recapture also identifies habitat-level patterns including the tendency for *T. basalis* to disperse as far as 75 m and to weedy areas rather than orchards and field crops [29]. Variation in dispersal distance and host parasitism between species and habitats demonstrates the need for *T. japonicus* specific data.

### 1.2. Winter Survival as a Condition for Successful Classical Biological Control

Since Platygastridae reproduction coincides with susceptible host stages [27], overwintering near host populations increases the probability for establishment from single or multiple release events. Woody vegetation where *H. halys* oviposits could be suitable habitat for *T. japonicus* release. Bark of woody trees is an overwintering habitat for *Telenomus*, a closely related genus of Platygastridae [37,39]. Female Platygastridae tend to diapause as adults [40,41], which leads us to assume that *T. japonicus* spend winter in the same life stage. Information about overwintering habitats would identify suitable locations for *T. japonicus* establishment and survival while *H. halys* are in reproductive diapause. *Trissolcus japonicus* survive temperatures as low as −17 °C [42], making establishment possible in temperate North American areas. Oregon’s mild winters and comparable climate to the parasitoid’s native range of China, Japan, and South Korea improve the potential for multi-year populations that will establish after initial redistributions.

*Trissolcus japonicus* dispersal, host-location, and overwintering success will determine suitability for continuation of parasitoid–host dynamics in an introduced area. Through strategic *T. japonicus* redistributions, we investigated (1) how well the parasitoid survives the period of release and initially detects sentinel *H. halys* egg masses and (2) dispersal distance after release in two perennial crops, and (3) the plant material conducive to successful overwintering. The outcome of this study will determine the optimal conditions for an adventive parasitoid to contribute to the biological control of the invasive *H. halys*.

## 2. Methods

### 2.1. Redistribution and Short-Distance Recovery

To assess short-term survival in anticipation of *T. japonicus* redistribution across widespread geographic areas, we released 40–50 mated, <72 h old, adult *T. japonicus* (9:1 F:M sex ratio) at 22 sites in Oregon (USA). Redistribution sites included four eco-regions, the Willamette Valley, Columbia Plateau, Eastern Cascades Slopes, and Rogue Valley. Each region differs in elevation and dominant tree cover and has a unique climate. All redistributed *T. japonicus* originated from adventive populations collected in Portland, Oregon (USA) and reared in a laboratory. Extensive statewide sampling for *T. japonicus* in the previous 3 years did not detect adventive wasps anywhere but the Portland metropolitan area [24,43]. Sites included urban habitats (N = 9) and areas adjacent to orchards (N = 13) in regions with known *H. halys* crop feeding damage.

We released wasps twice at 15 sites and once at 7 sites, from 15 May–30 June and 8 July–30 Aug 2017. To evaluate short-distance recovery, we placed 3 sentinel *H. halys* egg masses on *H. halys* host plants 5–10 m from the point of release. At least one egg mass at each site was freshly laid; the rest were frozen and <2 months old. Eggs frozen for less than 6 weeks may have parasitism rates reduced by as much as 50% but remain suitable for *T. japonicus* development [25]. During the second redistribution period, we did not have enough *H. halys* egg masses to place 3 egg masses at every site. Therefore, we also used yellow sticky cards (Alpha Scents: West Linn, OR, USA) to sample for *T. japonicus*. The cards and eggs were affixed to foliage of *H. halys* host plants, including *Acer*, *Catalpa*, *Ilex*, and *Platanus*. After placing sentinel egg masses or yellow sticky cards at each site, we released wasps by gently tapping them from rearing cups onto the foliage of *H. halys* host plants. After 3 days, we collected sticky cards and eggs. We placed 170 sentinel *H. halys* egg masses and recovered 148. Recovered egg masses were transported to a growth chamber set to 23 °C (16:8 L:D), and we recorded the number of emerged wasps and the species. Frozen eggs from which adult wasps did not emerge after 5 weeks were dissected for the presence of incompletely developed wasps.

In 2018, one year after wasps were released, we revisited all but one site to search for overwintered *T. japonicus*. Twice per summer, we placed 3 yellow sticky cards per site on *H. halys* host plants at a height of 1.5 m. On trees where we placed sticky cards and sentinel egg masses, we also searched for wild *H. halys* egg masses. Cards remained on trees for two weeks. When available, we supplemented sampling with sentinel *H. halys* egg masses (N = 90), leaving them at sites for 3–4 days before returning them to a lab to evaluate parasitism. Since each release site was at least 10 km away from known recovery sites of adventive *T. japonicus*, we attributed recoveries in the following year to the previous season’s redistributed wasps. Mark-recapture work from another study failed to detect parasitoids beyond 75 m from the release site [29], which supports the attribution of wasps recovered at short distances to the previous season’s redistribution.

### 2.2. Releases and Dispersal in Crops

We investigated *T. japonicus* dispersal and host-location at two raspberry fields (cv. ‘Meeker’) at Oregon State University Research stations and two commercial hazelnut orchards (cv. ‘Yamhill’ and ‘Dorris’). Each commercial site was 2–4 ha. Raspberry fields were 100 km apart, and the hazelnut orchards were 14 km apart. The fruit of both crops are suitable hosts for *H. halys*, and each plant provides a contrast in canopy shape and shaded area. Raspberry bushes grow compactly in rows, and space between plant rows (1.8–2.4 m) receives direct sunlight. Hazelnuts are a deciduous tree that can grow up to 9 m, and space between rows (3–4.5 m) is shaded in the summer. At each site, we released 40–50 adult, <72-h old *T. japonicus* (9:1 F: M sex ratio) on partly sunny to sunny mornings, twice per year in 2017 and 2018, between 15 June and 20 August. In 2017, we released wasps on the foliage of a tree or bush along the field perimeter (edge) and on the foliage of a plant 100 m inside hazelnut and 30 m inside raspberry (interior). Three transects were established per site for edge and interior releases. At edges, we set up sentinel fresh (N = 22) or frozen (N = 185 <40 days old) *H. halys* egg masses at the following distances: 5, 10, 20, 30, 40, and 50 m (N = 6 per release) from the point of release. In crop interiors, we set up egg masses at 5, 15, and 25 m (N = 6 per release) from point of the release in raspberry and at 10, 20, 40, 60, and 80 m (N = 10 per release) from the point of release in hazelnut. The same number of egg masses were placed at each distance at every site. Eggs were placed at a height of 1.5–1.8 m in mature hazelnut trees and at approximately 1.2 m in raspberry. No insecticides or herbicides were applied the week prior to releases through recovery of egg masses or sticky cards.

In 2018, the experiment was repeated at the same sites but with some changes. One week before releasing wasps in June 2018, we placed 5 yellow sticky cards at the perimeter of each site to determine if previously released *T. japonicus* may have overwintered. None were found. Only yellow sticky cards were used at all distances to measure dispersal. Due to a shortage of available *H. halys* eggs, fresh eggs (N = 29) were placed only at a subset of distances (5, 10, and 50 m). Moreover, as in 2017 parasitism rates by distance were not significantly different in interior and edge releases (χ = 0.40, *p* = 0.52), in 2018 parasitoids were released only at field edges. Therefore, analyses of *T. japonicus* at each distance was pooled for interior and exterior releases. In both years, yellow cards or egg masses were collected 72 h after releases.

### 2.3. Parasitoid Longevity and Fecundity in Overwintering Habitat

**Figure 1:**
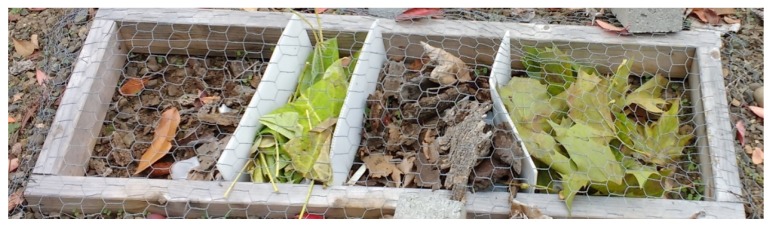
Outdoor overwintering structures filled with leaves or bark. Vials containing *T. japonicus* were placed beneath each section of plant material.

We constructed six 1.1 × 0.3 m wooden structures to evaluate *T. japonicus* survival in outdoor winter conditions. In October 2017, we placed 3 structures at the North Willamette Research and Extension Center (Aurora, OR, USA) and 3 at the Oak Creek Urban Horticulture Center (Corvallis, OR, USA) on the ground in partially shaded semi-natural habitats. Each structure was subdivided into sections filled with plant material from one of four treatments: catalpa leaves, catalpa bark, sycamore leaves, or sycamore bark (Figure 1). Each treatment was separated by Coroplast (Plaskolite: Columbus, OH, USA) dividers, and the structures were covered with poultry netting for rodent protection. In addition to the two outdoor locations, we placed 5–6 replicates of each overwintering woody material in a low-temperature cabinet (Intellus: Perry, IA, USA) to control for the effects of outdoor conditions on wasp survival.

All wasps evaluated for winter survival originated from field-collected populations in Portland, OR (USA). In 2017, five mated, female *T. japonicus* wasps were placed within 5.1 cm diameter foam clip cages. Due to high moisture penetration and mortality in foam clip cages, we switched to plastic scintillation vials (Fisher Scientific: Pittsburgh, PA, USA) in 2018 and only used wasps that were <72 h from emergence. We placed each of the four plant materials inside every clip cage or scintillation vial, set these in the outdoor wooden structures, and laid 2.5 cm of each woody treatment above the wasps. Vials or clip cages were sealed with a fine mesh to prevent escape. Each outdoor structure and indoor growth chamber had a similar number of vials or clip cages containing the four plant materials (Table 1). Temperatures in the growth chamber were changed weekly to represent the same outdoor conditions from a representative weather station in Corvallis, OR (AgriMet crvo; 44.63416 N, −123.19 W).

We assessed wasp mortality in each clip cage or vial every two weeks starting 28 October 2017 and 18 October 2018. In 2017, the experiment lasted for 12 weeks, until the final wasp died, and in 2018, we terminated the experiment after 16 weeks in outdoor sites and 20 weeks in the growth chamber. We brought vials with surviving wasps to a laboratory growth chamber set to 20 °C and provided surviving wasps with fresh and frozen *H. halys* egg masses to evaluate F1 progeny. We evaluated longevity in wasps that survived winter, recorded mortality every 3–5 days, and provided them with a honey-water solution.

### 2.4. Data Analysis

#### 2.4.1. Redistribution and Short-Distance Recovery

At sites with redistributed wasps, we compared the percentage of locations where *T. japonicus* were recovered after 3 days and in the following year. Since most of the 2018 samples involved yellow sticky cards, data were classified according to their presence or absence, and we compared recoveries between years using a chi-square test.

#### 2.4.2. Releases and Dispersal in Crops

We compared the number of emerged wasps, parasitized eggs (emerged + unemerged wasps inside eggs), and predation at different distances from the release point. We excluded 10 egg masses in raspberry where all eggs were missing due to abiotic factors. Since the number of eggs differed slightly between individual egg masses, we used proportional data. We used Generalized Linear Mixed Models with penalized quasilikelihood, distance as a fixed effect, and site as a random effect to compare predation and parasitism. As *H. halys* is a perimeter pest in multiple crops [44,45], we also compared the three outcomes of sentinel egg masses (emerged wasps, parasitized eggs, and predation) of damage by pooling data between close (5–15 m, N = 118) and further distances (20–50 m, N = 118) from the point of release. Dunnett’s pairwise comparisons were used as a post-hoc test to compare predation and parasitism between the point of release and more distant locations. Data from yellow sticky cards were not normally distributed. Therefore, we used a Kruskall–Wallis test to compare the number of *T. japonicus* collected on yellow cards at each distance in 2018. Low *T. japonicus* recapture in hazelnut prevented statistical analysis, and we only present a summary of results.

#### 2.4.3. Overwintering Habitat

At the conclusion of the winter period, we compared differences in percent survival with a factorial ANOVA using site, plant, and material as dependent variables. We also compared the average number of weeks until wasps died in each treatment with an ANOVA. Kaplan-Meier survival curves were used to evaluate the rate at which *T. japonicus* died between treatments pooled across sites, in outdoor sites, and in the indoor growth chamber. To determine if wasp survival was affected by plants or woody debris treatments between the growth chamber and outdoor sites, we used log-rank tests with a Bonferroni correction. In replicates with wasps that survived winter, we provided *H. halys* egg masses. We compared how parasitism rates differed between overwintering treatments using ANOVA and how sex ratio differed between treatments using generalized linear models with binomial family. Additionally, we used a one-sample t-test to compare parasitism rates by overwintered wasps to the median parasitism rate of recently emerged wasps on fresh and frozen *H. halys* egg masses in a related study [25], which corresponded to 92% and 27%, respectively. We evaluated for differences in longevity, until the 80th day after ending winter, of surviving wasps between plant materials using log-rank tests with a Bonferroni correction and in parasitism by plant materials with a Kruskall Wallis Test. These analyses used the survival package in R 3.3.1 [46].

## 3. Results

### 3.1. Redistribution and Short-Distance Recovery

**Figure 2:**
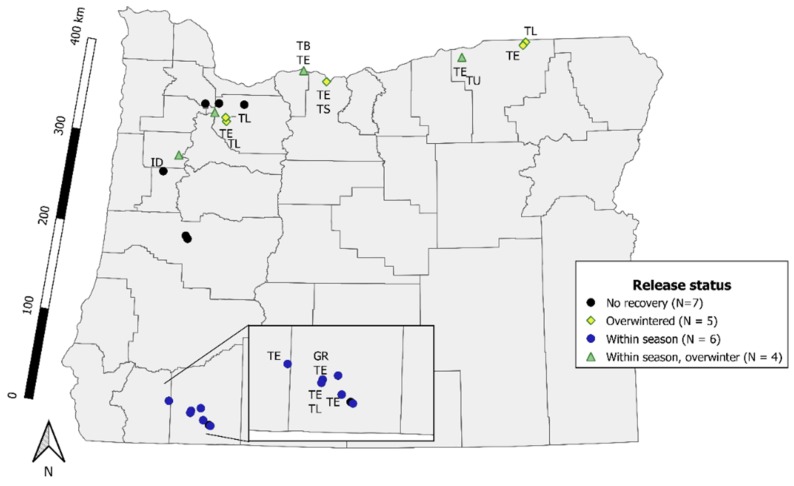
Oregon (USA) locations of *T. japonicus* redistribution and evaluation for short-term recovery and release status. Release status refers to the classification of wasps never being recovered after 3 days or 1 year (No recovery), being recovered after three days (Within season), in the following season (Overwintered) and both after three days and in the following season (Within season, overwinter). Abbreviations next to sites indicate native Platygastridae detected on yellow sticky cards or emerged from sentinel *H. halys* eggs. TB = *Trissolcus brochymenae*, TE = *T. euschisti*, TS = *T. strabus*, TU = *T. utahensis*, TL = *Telenomus*, GR = *Gryon*, ID = *Idris*.

Three days after releasing parasitoids, we recovered egg masses parasitized by *T. japonicus* at 10 of 22 release sites across Oregon (Figure 2). At seven sites, we recorded parasitism and successful development from frozen *H. halys* egg masses by three native parasitoid species, *Trissolcus euschisti* (Ashmead), *Trissolcus utahensis* (Ashmead, 1893), and *Anastatus* spp. *Trissolcus euschistii* also emerged from a freshly laid sentinel egg mass at one of the same sites in southern Oregon.

In 2018, the year after release, *T. japonicus* emerged from six *H. hays* egg masses (two sentinel and four wild) at a single site. We detected *T. japonicus* on yellow sticky cards at nine sites (Figure 2). These included four sites where we did not detect wasps in the initial year. Seven native Platygastidae species were also recovered on yellow sticky cards, and most were found at the same sites where *T. japonicus* survived the winter. We recovered *T. euschisti*, on cards or eggs at 12 sites where we released *T. japonicus*. *Trissolcus japonicus* were recovered at a similar number of sites after three days and after one year (χ^2^ = 0, *df* = 1, *p* = 1).

### 3.2. Dispersal in Crops - Raspberry

**Figure 3:**
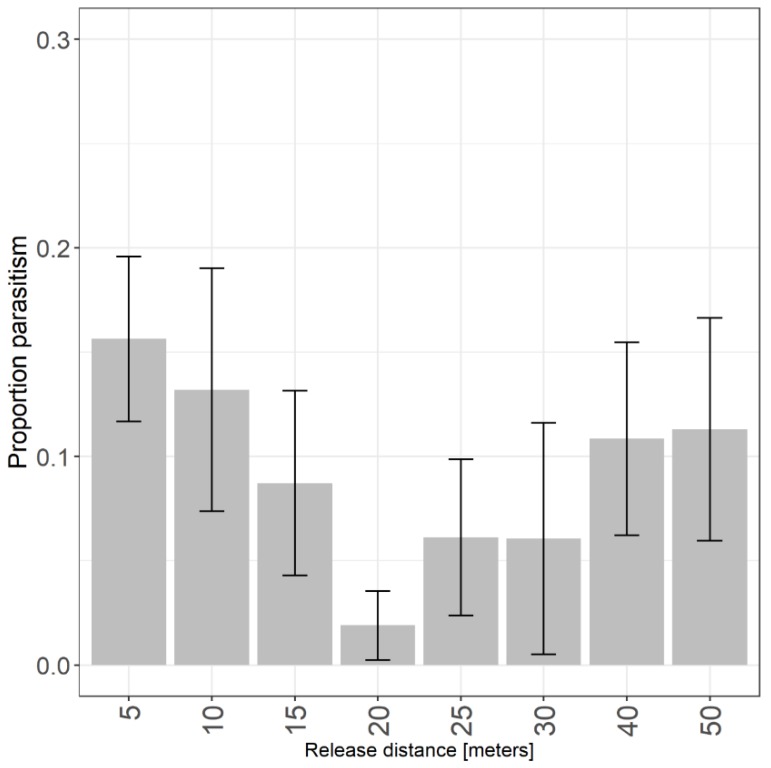
Proportion of *H. halys* eggs parasitized by *T. japonicus* by distance from release in raspberry.

In both years, we recovered parasitized egg masses at all distances from the release point in raspberry, and there was no difference in parasitism by distance (χ^2^ = 6.12, *df* = 8, *p* = 0.63; Figure 3). Mean parasitism (± SE) was greatest at 5 m (15.6 ± 4.0%) and 50 m (13.7 ± 6%), the closest and farthest distances to the point of release. When pooling shorter distances (5–15 m; N = 118) and farther distances (20–50 m; N = 118), parasitism, which includes emerged and unemerged wasps, was higher at points closer to the release (mean ± SE: 13.5 ± 2.7%). However, this was not significantly different from distances beyond 20 m (mean ± SE: 7.5 ± 0.2 %; χ^2^ = 2.54, *df* = 1, *p* = 0.11). *Trissolcus japonicus* were recovered on 22% of yellow cards (N = 8), and there was no difference in recovery by distance from release (χ^2^ = 5.51, *df* = 5, *p* = 0.36). When pooling the presence of *T. japonicus* on cards and eggs there was no difference in recovery by distance from release (χ^2^ = 7.08, *df* = 7, *p* = 0.53). Mean predation was below 10% at all distances, and there was a trend towards greater predation away from the point of release (χ^2^ = 15.1, *df* = 7, *p* = 0.03). Pairwise comparisons indicated marginally greater predation at 25 m (*z* = 2.74, *p* = 0.04) and 50 m (*z* = 2.67, *p* = 0.04) compared to 5 m.

### 3.3. Releases in Crops-Hazelnut

Of 177 eggs placed in hazelnut, only four were parasitized by *T. japonicus*–at distances of 0, 10, and 40 m from the point of release. A single *Telenomus podisi* Ashmead, 1893 was observed guarding an egg mass, but no wasps emerged. Of 61 yellow sticky cards placed in hazelnut, *T. japonicus* was found on three yellow cards at 5, 10, and 40 m. A native Platygastridae, *T. euschisti*, was found on eight cards.

### 3.4. Parasitoid Longevity and Fecundity in Overwintering Habitat

**Figure 4:**
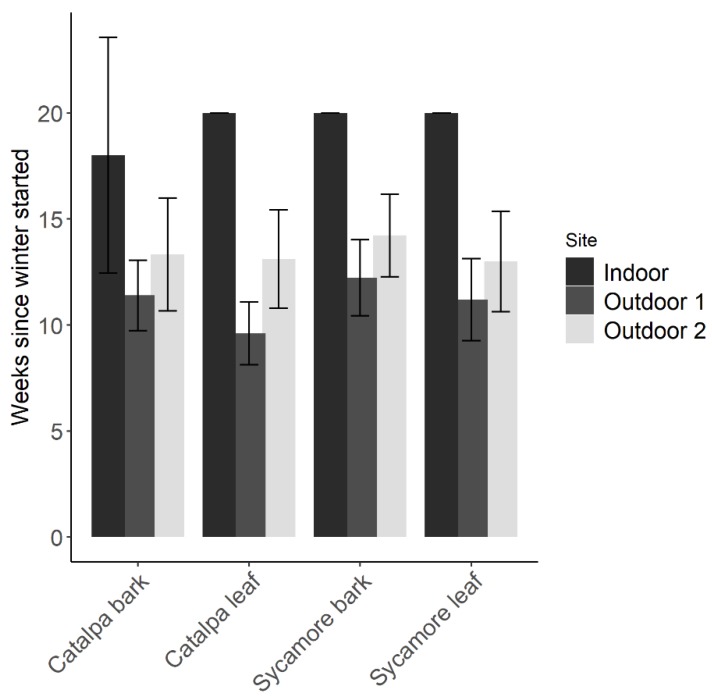
Number of weeks (Mean ± 95% CI) that adult parasitoids survived winter storage in leaf and bark debris.

At the conclusion of winter storage, a similar number of wasps survived each plant material However, a greater proportion of wasps survived inside a growth chamber with simulated winter temperatures (*F*_2,83_ = 45.2, *p* < 0.01). Minimum temperatures in each outdoor site were −3.9 °C and −1.7 °C. Maximum temperatures at the outdoor sites were 18.6 °C and 17.1 °C. After 20 weeks in a growth chamber, approximately 50% of wasps remained alive. Wasps at the outdoor sites died or were removed after 16 weeks in 2018. The average number of weeks until the final wasp died differed only by site (*F*_2,83_ = 73.78, *p* < 0.01) rather than plant material (*F*_3,83_ = 1.40, *p* = 0.24). In all plant materials, there were often surviving wasps after 20 weeks in the indoor environment, while the last wasp in each outdoor replicate often died within 9–16 weeks (Figure 4).

**Figure 5:**
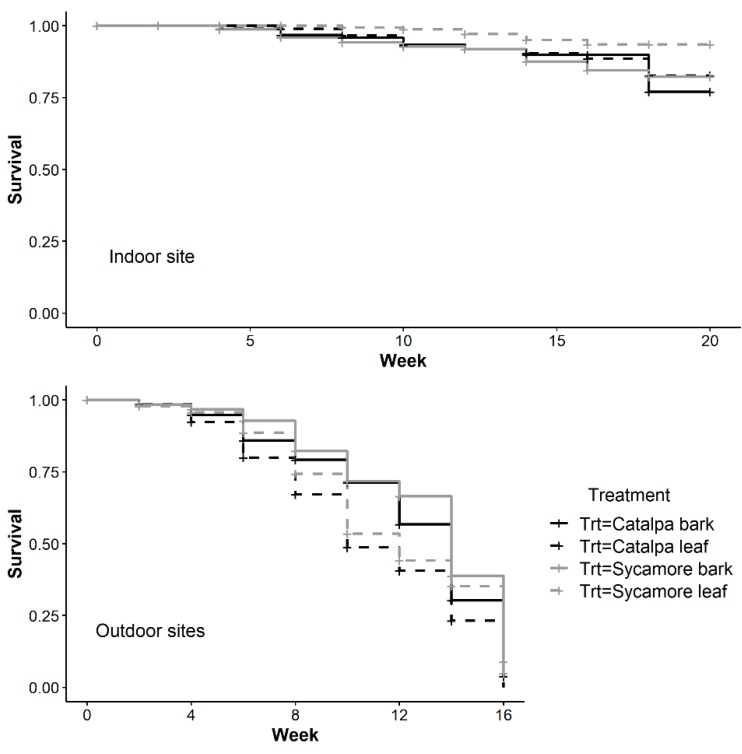
Kaplan-Meier survival curve indicating *T. japonicus* survival over time during winter in a single indoor growth chamber and two outdoor sites.

The rate at which wasps died over time differed between treatments in outdoor, indoor, and all sites (Table 2). In the indoor growth chamber, survival over time was greater in sycamore leaves than catalpa bark (*p* = 0.05) or sycamore bark (*p* = 0.02). In both outdoor sites, survival over time was greater in catalpa bark than leaves (*p* = 0.01), and in sycamore bark compared to catalpa leaves (*p* < 0.0001). In the outdoor sites, *T. japonicus* adult mortality increased at a similar rate across treatments until approximately week six, when more wasps began dying in leaves (Figure 5). In the indoor growth chamber with winter temperatures, we found the opposite pattern of greater wasp survival over time in leaves. Survival of *T. japonicus* in sycamore leaves and bark was greater over time in the outdoor sites (*Z* = 2.52, *df* = 1, *p* = 0.01) but not in the indoor growth chamber (*Z* = 1.16, *df* = 1, *p* = 0.24)

Pooled across indoor and outdoor sites, twenty-nine replicates had surviving *T. japonicus*; five catalpa leaf and sycamore leaf, nine sycamore bark, and 10 catalpa bark. We provided 30 egg masses to replicates with surviving *T. japonicus* (catalpa bark N = 10, catalpa leaf N = 5, sycamore bark N = 10, sycamore leaf N = 5). Mean (± SE) parasitism rates (%) from fresh (65.2 ± 11.8, N = 9) and frozen (59.3 ± 7.4, N =21) *H. halys* egg masses were similar (*F*_1,221_ = 0.19, *p* = 0.67). Though sex ratio (F:M) in frozen egg masses (0.59 ± 0.09) was lower than from fresh egg masses (0.76 ± 0.04), high variability in sex ratio (range: 0–0.95) led to a comparable gender balance between egg types (*z* = 0.83, *p* = 0.40). The plant material in which wasps were stored had no effect on sex ratio (χ^2^ = 0.69, *df* = 3, *p* = 0.87). Mean (± SE) parasitism rate from wasps stored in catalpa bark (73.3 ± 8.9 %, N = 10) and sycamore leaves (70.6 ± 7.5, N = 5) were highest, and the lowest mean parasitism rate was from wasps stored in catalpa leaves (42.0 ± 17.1, N = 5). Nonetheless, small sample sizes in each woody material contributed to comparable parasitism rates from the overwintered wasps (χ^2^ = 2.22, *df* = 3, *p* = 0.53). In wasps that survived winter, there was a difference in longevity over time between plant material (χ^2^ = 25.00, *df* = 3, *p* < 0.001). On average (± SE), wasps survived the greatest number of days in catalpa leaves (59.6 ± 13.8) and sycamore bark (48.0 ± 7.5), followed by sycamore leaves (49.2 ± 14.0) and catalpa bark (27.8 ± 3.8).

## 4. Discussion

Redistributing an egg parasitoid was expected to hasten its establishment and increase parasitism of the invasive *H. halys*. In three Oregon eco-regions, *T. japonicus* adults successfully survived a release, located host egg masses, and were detected following a winter. Wasps overwintered in shrub steppe climates with average winter lows of −3 °C to cool summer Mediterranean climates with average winter lows of 0.9 °C. Minimum low temperatures at these sites are at least 10 °C above *T. japonicus* mortality thresholds [42], making Pacific Northwest climates suitable for colonization. The shared factor in most sites with adventive *T. japonicus* is urban areas or proximity to ornamental trees. Urban residential gardens often contain herbaceous flowering forbs, providing nectar that enhances parasitism [47]. Mobile insects experience reduced pesticide contamination in urban gardens compared to agriculture [48]. As *T. japonicus* has a high mortality rate from broad-spectrum insecticides applied to manage agricultural pests [49], the parasitoid will further benefit from its ability to colonize urban areas.

### 4.1. Parasitoid Release and Dispersal Assessment

*Trissolcus japonicus* dispersed to *H. halys* egg masses or yellow sticky cards at least 50 m away from release sites, a comparable dispersal distance to other small-bodied parasitoids [50,51]. Some work indicates no preference in direction or distance when *T. basalis* searches for host eggs within a 12–50 m transect [35,50]. On the other hand, other studies demonstrated higher Pteromalidae parasitism 5 m away from release [51] and 14× greater *Ooencyrtus submetallicus* parasitism of *N. viridula* egg masses at 1 m away from release [35]. Though there was no significant difference in dispersal distance from the release site, low parasitism rates in crop interiors indicate avoidance of this area. Other agricultural systems show greater parasitism adjacent to parasitoid release sites [52,53], and this is logical given limited parasitoid dispersal capacity. Surprisingly, the next highest parasitism rate occurred at 50 m, the farthest point from the release site. Following dispersal to greater distances, some parasitoids experience increased oviposition rates [54], which suggests the energetic costs of movement do not result in a direct decrease in reproduction. While host location behaviors will vary between parasitoid species, our results indicate that *T. japonicus* travels variable distances for host-location.

While a maximum recovery distance of 50 m is unlikely to include all areas within an orchard, three factors make for a conservative estimate for spread. First, *T. japonicus* population size was low in releases. Mass rearing of >10,000 individuals is recommended for biological control programs [55]. Limited availability of *H. halys* egg masses constrained population sizes for this experiment, and a ten-fold increase in *T. japonicus* release population is more realistic to reduce *H. halys* populations at a field-scale. Second, the perimeter foraging patterns of *H. halys* cause most adults to lay eggs in outer orchard rows, which are within the 50 m recovery distance. Habitat preferences can be taxonomically specific, as *Anastatus* parasitism on *N. viridula* and *H. halys* was elevated in woodlands [56,57]. Several *Trissolcus* species disperse readily between woodlands and crops [57]. Like *H. halys*, *T. japonicus* may prefer edge habitat. Finally, our maximum recapture distance of 50 m does not preclude the possibility that *T. japonicus* travel farther in search of host eggs. We acknowledge a marginal chance that parasitized egg masses could be attributed to adventive wasps not previously detected, but several factors make this scenario less likely. We sampled *T. japonicus* extensively in Oregon since 2014 and initially detected the parasitoid in 2016 [24] at locations no closer than 30 km to experimental release sites. While setting up sentinel egg masses and yellow cards, we searched and did not find any wild-laid *H. halys* egg masses, which would sustain adventive *T. japonicus* populations. Low availability of the suitable host life stage meant that sentinel egg masses were likely the sole host sources within sites.

While hazelnut’s broadleaf canopy benefits the shade-adapted *T. japonicus* [13], our expectation of greater parasitism in hazelnut was not supported. Raspberry’s compact foliage and greater exposure to heat and sunlight may limit *T. japonicus* dispersal to crops with more shade. Insecticides are not the cause for low dispersal in hazelnut, since none were applied within three weeks of release. Sheer volume of foliage surface area in hazelnut orchards may affect foraging and dispersal patterns that could limit dispersal distances of the wasp. Platygastrid parasitism rates also average below 10% in other orchard crops [29,58]. By restricting sentinel egg or yellow card placement at heights below 1.8 m, we may underestimate parasitism rates, which are highest in the mid-canopy, at heights of approximately 4.3 m [59]. Equipment to detect wasps at heights above head level may be necessary for better interpretation of *T. japonicus* dispersal in orchard crops. The challenge of quantifying parasitism rates in deciduous trees limits inferences about host-tracking beyond and within orchard boundaries.

### 4.2. Parasitoid Longevity and Fecundity in Overwintering Habitat

Decomposition and exposure to precipitation make leaf litter a poor parasitoid overwintering habitat relative to bark. The initial weeks of winter storage included comparable *T. japonicus* survival between leaf litter and bark and temperatures above 9 °C, a threshold for which Platygastridae remain active [60]. As precipitation arrived and daily temperatures cooled, survival decreased more quickly in catalpa and sycamore leaf litter at outdoor sites. We also observed slugs and detritivores feeding on leaf litter to the extent that only the stem of catalpa leaves remained after 8 weeks. Upon inspecting bark in the field and in growth chambers, we regularly observed wasps nestled at least 1 cm within bark. Bark protects wasps from precipitation and provides insulation with temperatures several degrees above air temperature [61,62]. Wasps overwintering within trees, rather than our artificial structures, would be further sheltered from air temperature. Increased survival in sycamore leaves in growth chambers was probably an artifact of an environment lacking precipitation. Remaining dry is critical to prevent ice crystallization on the exoskeleton. One implication of wasps overwintering in bark is that spring soil and weed management including mechanical flailing [63] will not be lethal for *T. japonicus*.

Reproduction from overwintered *T. japonicus* was lower than reproduction from lab-reared colonies [25] yet surviving wasps parasitized eggs independent of overwintering plant material. Wasps that survived winter in leaf litter, an overwintering habitat with quicker mortality, oviposited in host egg masses at a comparable rate to wasps stored in bark. Frozen egg masses did not affect F1 *T. japonicus* emergence, which was double the expected rate from three-month-old frozen *H. halys* egg masses [25]. When overwintered Platygastridae are supplemented with honey, there is increased success of oviposition into host eggs [64,65]. This suggests that *Trissolcus* search for extrafloral nectaries or other energy sources on warmer winter days during winter. However, another study that provided honey to naturally overwintered *T. semistratus*, showed fewer offspring from overwintered females [66]. A male-biased sex ratio from some offspring may be attributed to females that did not mate before transfer to an overwintering environment but also occurs after cold-storage in some parasitoids [67]. The Pacific Northwest’s period of *H. halys* egg-laying between May and October means that *T. japonicus* may be in diapause for as few as five months. Longer winter periods increase mortality and minimize dry weight or energy, which will have consequences for reproductive capacity [68,69]. Since *T. japonicus* overwinters as an adult, it can search for resources immediately after emergence and sustain itself on nectar or water when host eggs are scarce in the spring.

Post-winter storage *T. japonicus* longevity of greater than 30 days coincides with the onset of egg production by overwintered *H. halys* in western Oregon. This synchrony with *H. halys* emergence provides a positive outlook for long-term impact on *H. halys* populations. Some wasps survived at least 220 days from emergence, confirming that *T. japonicus* recovery after one year at redistribution sites is likely due to the survival of redistributed populations. Platygastridae stored at 15 °C for 120–200 day diapause periods survive an additional four to six weeks afterwards [70], a comparable duration to our experiment. *Trissolcus japonicus* stored in outdoor catalpa leaves experienced the highest mortality in winter, while wasps subjected to the laboratory simulated environment lived longest. Though survival longevity after winter storage differed statistically between treatments, the small sample size of each treatment—5–10 each—limits inferences about the effect of winter environment on post-winter longevity. Winter temperatures may have selected against weaker adults in each treatment, making all surviving *T. japonicus* equally likely to persist for an additional 25–50 days after re-acclimation to warmer temperatures. It will be necessary to evaluate post-winter fitness in climates with longer winter periods, as the energetic costs of lipid depletion reduce fitness directly or through poorer reproduction [71].

## 5. Conclusions

This study represented an initial attempt to identify establishment patterns in a minute parasitoid used for biological control of an invasive species. Egg parasitoids will disperse amidst managed habitats, but low population size of the released cohorts restricts our ability to identify the release density necessary for an economic impact. A 40% parasitism rate is possible with release of 10,000 *Trissolcus basalis* in a 25 m^2^ area [29], and the 20% parasitism rate in raspberry is a positive indication that *T. japonicus* will have widespread effects on its target host when redistributed in larger populations. Discovery of *T. japonicus* alive after winter in plant material and one-year post-release confirms that Platygastridae overwinter as adult females [26,40]. Parasitoids introduced to a novel habitat need an abundant supply of their host insect to persist. In addition to needing a host, we demonstrate the relevance of bark for overwintering Platygastridae and their capacity to disperse and search for host eggs at least 50 m from release sites. The introduction of a non-native parasitoid could affect the community of native congeneric parasitoids in European [72] and North American [73] sites where *H. halys* is present. This will require sampling native wasp populations in future years. Invasive species pose challenges in their introduced environment, and biological control with a co-evolved parasitoid is a possible management strategy in climates comparable to the native range.

## Figures and Tables

**Table 1:** Number of replicates where *T. japonicus* experienced winter conditions in Oregon (USA) winters. Values in parentheses indicate additional number of replicates placed inside growth chambers with simulated winter temperatures.

	2017		2018	
	Bark	Leaves	Bark	Leaves
Catalpa	24 (10)	24 (8)	19 (5)	19 (5)
Sycamore	24 (8)	24 (8)	18 (6)	18 (5)

**Table 2:** X^2^ (Treatment) and Z scores (plant and woody debris) from log-rank tests comparing *T. japonicus* survival distributions at all overwintering sites, the two outdoor sites, and single indoor environmental growth chamber. ** indicates Bonferroni-corrected *p* < 0.01, * indicates *p* < 0.05.

Variable	All Sites	Outdoor	Indoor
Treatment	13.86 **	23.00 **	10.01 *
Plant	3.70 **	2.52 **	1.16
Woody debris	0.326	4.02 **	2.36 *
